# Clinical and molecular assessment of an onco‐immune signature with prognostic significance in patients with colorectal cancer

**DOI:** 10.1002/cam4.4568

**Published:** 2022-02-09

**Authors:** Pankaj Ahluwalia, Ashis K. Mondal, Meenakshi Ahluwalia, Nikhil S. Sahajpal, Kimya Jones, Yasmeen Jilani, Gagandeep K. Gahlay, Amanda Barrett, Vamsi Kota, Amyn M. Rojiani, Ravindra Kolhe

**Affiliations:** ^1^ Department of Pathology Medical College of Georgia at Augusta University Augusta Georgia USA; ^2^ Department of Neurosurgery Augusta University Augusta Georgia USA; ^3^ Department of Molecular Biology and Biochemistry Guru Nanak Dev University Amritsar India; ^4^ Department of Medicine Medical College of Georgia at Augusta University Augusta Georgia USA; ^5^ Department of Pathology Penn State College of Medicine Hershey USA

**Keywords:** colon, colorectal cancer, gene signature, immune, personalized medicine, preventive, prognostic genes

## Abstract

Understanding the complex tumor microenvironment is key to the development of personalized therapies for the treatment of cancer including colorectal cancer (CRC). In the past decade, significant advances in the field of immunotherapy have changed the paradigm of cancer treatment. Despite significant improvements, tumor heterogeneity and lack of appropriate classification tools for CRC have prevented accurate risk stratification and identification of a wider patient population that may potentially benefit from targeted therapies. To identify novel signatures for accurate prognostication of CRC, we quantified gene expression of 12 immune‐related genes using a medium‐throughput NanoString quantification platform in 93 CRC patients. Multivariate prognostic analysis identified a combined four‐gene prognostic signature (*TGFB1*, *PTK2*, *RORC*, and *SOCS1*) (HR: 1.76, 95% CI: 1.05–2.95, *p < 0.02). The survival trend was captured in an independent gene expression data set: GSE17536 (177 patients; HR: 3.31, 95% CI: 1.99–5.55, *p < 0.01) and GSE14333 (226 patients; HR: 2.47, 95% CI: 1.35–4.53, *p < 0.01). Further, gene set enrichment analysis of the TCGA data set associated higher prognostic scores with epithelial–mesenchymal transition (EMT) and inflammatory pathways. Comparatively, a lower prognostic score was correlated with oxidative phosphorylation and MYC and E2F targets. Analysis of immune parameters identified infiltration of T‐reg cells, CD8^+^ T cells, M2 macrophages, and B cells in high‐risk patient groups along with upregulation of immune exhaustion genes. This molecular study has identified a novel prognostic gene signature with clinical utility in CRC. Therefore, along with prognostic features, characterization of immune cell infiltrates and immunosuppression provides actionable information that should be considered while employing personalized medicine.

## Graphical abstract

Colorectal cancer has a complex tumor microenvironment that also provides a unique opportunity for the molecular classification of these patients. In this study, we have used NanoString technology to quantify potentially prognostic mRNAs in the Augusta University cohort. Further, a similar prognostic pattern was observed in the TCGA data set.

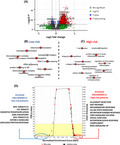

## 1 INTRODUCTION

Colorectal cancer is the most common digestive cancer with approximately 53,000 annual deaths in 2020 in the United States.^1^ Although surgery is curative for 15%–20% of eligible CRC patients, recurrence is the unfortunate outcome for most of this resected patients.^2^ Inherent molecular heterogeneity of CRC tumors leading to differential susceptibility to chemotherapy and immunotherapy are significant barriers to reducing the overall mortality. There is a need to identify prognostic and predictive tools that can stratify patients based on mortality risk and response to therapies, respectively. Prognostic gene signatures with underlying immune perturbations may assist in the stratification of patients for emerging personalized therapies such as NK cell‐based therapies or a combination of chemotherapy and immunotherapy.^3^, ^4^, ^5^

Recently, gene expression‐based classification has helped in the characterization of several cancers with varying degrees of validation.^6^, ^7^, ^8^ In CRC, one of the most robust classifiers is based on consensus molecular subtypes (CMSs) but its application is limited due to its dependence on >500 genes and the underlying complexity of the method.^9^ There is a need to identify cost‐effective clinical assays for the prognosis of CRC patients.^10^ Recent advances in RNA‐sequencing technologies and microarray have been widely utilized to identify prognostic gene signatures. Gene signatures based on immune and lipid mediator pathways have recently been found to be prognostic in CRC patients.^11^, ^12^ Although several gene expression biomarkers have been identified, they lack clinical validation. Thus, there is a need to identify gene signatures with prognostic significance and associated pathway perturbations that can stratify patients based on survival and assist in the application of personalized therapeutics. In this study, we have identified a novel four‐gene signature with prognostic and clinical utility in CRC. The expression of 12 immune‐related genes was quantified in FFPE tissues of CRC patients and was analyzed in the context of public data sets. Network analysis and gene set enrichment analysis of colorectal cancer patients identified significant perturbations of homeostatic functions in the high‐risk group. Deconvolution analyses revealed higher infiltration of Neutrophils, B cells, and macrophages in high‐risk patients. Further, there was higher infiltration of CD8^+^ T cells but these tumors were also found to be enriched in immune exhaustion genes.

## 2 MATERIALS AND METHODS

### 2.1 Design of gene panel and data acquisition

We accessed scientific literature using PubMed to identify 12 genes with an immunological role in colorectal cancer (Table 2). The TCGA‐COAD data set was analyzed for genetic mutation, transcriptome, and other clinical variations.^13^, ^14^ The mutation data were visualized through the “maftools” package in the R program and Gene set cancer analysis.^14^, ^15^ For external validation, gene microarray data and corresponding clinical information of verifying cohorts GSE17536 and GSE14333 were downloaded from the GEO database (https://www.ncbi.nlm. nih.gov/geo/).^16^, ^17^, ^18^

### 2.2 Patient samples

The study included 250 FFPE blocks of colorectal cancer patients with the protocol approved by the Institutional Review Board (IRB‐HAC # 611298) from the Medical College of Georgia at Augusta University. As there were multiple blocks of each patient, only blocks with high‐tumor content as assessed by a pathologist were included in this study. The samples with incomplete documentation, lack of tumor tissue in blocks, failure of RNA isolation, or degradation of RNA were excluded from this study.

### 2.3 RNA isolation from FFPE blocks

Five micrometers thick, parallel sections were generated from FFPE tissues. H&E staining was performed using a standard protocol and was examined for tumor‐rich regions (>50% tumor) by a board‐certified pathologist (RK). Macrodissection was performed to isolate RNA from selected regions. Total RNA was isolated through miRNEasy FFPE kit (Qiagen) using a standard protocol. The RNA was further quantified using a Nanodrop spectrophotometer (NanoDrop ND‐1000, NanoDrop Technologies).

### 2.4 Quantification of mRNA using NanoString platform

The quantification of mRNA molecules was performed using a digital quantification instrument (NanoString Technologies Inc.). nCounter PlexSet technology is a digital quantification system, which identifies and quantifies specific RNA molecules using a target‐specific oligonucleotide probe pair. The design and construction of probes were performed by NanoString and Integrated DNA Technologies, Inc. (IDT). The target sequences, Probe A, and Probe B sequences are present in Table S1. PlexSet consists of a unique fluorescently coded barcode linked to reporter tags and a biotinylated universal capture tag. The specificity of the signal is achieved through a unique barcoded signature. The fluorescence by reporter tags is processed during subsequent data capture and analysis. The universal capture tag anchors specific RNA molecules to the streptavidin‐coated lane on the nCounter instrument.^19^ The data collection was performed on the nCounter Digital Analyzer (DA). The field of view (FOV) setting for DA was set at 280, as previously noted.^20^ A total of 300 ng of total RNA was used as an input for this analysis. The raw gene expression counts were further processed and normalized using nCounter software.

### 2.5 Identification of prognostic gene signature

Univariate and multivariate Cox proportional hazard models were used to analyze clinicopathological variables. For every patient, the prognostic score was calculated by multiplying the expression value of a gene with its corresponding Cox proportion regression coefficient (prognostic score = Σ Cox regression coefficient of Gene_*i*_ × expression value of gene Gene_*i*_). Patients were stratified into low‐ and high‐risk groups according to the median risk score, and survival was assessed using the Kaplan–Meier method and the log‐rank test. The hazard ratios (HRs) and log‐rank *p* values were based on overall survival (OS). The combined prognostic gene signature was validated in two independent data sets GSE17536 (*n* = 177) and GSE14333 (*n* = 226).

### 2.6 Identification of differentially expressed pathways

DEseq2 package in R was utilized to identify differentially expressed genes (DEGs) (http://bioconductor.org/packages/release/bioc/html/DESeq2.html) between high‐risk and low‐risk patients in the TCGA data set. The results were graphed as a volcano plot using the “enhancedvolcano” package (http://bioconductor.org/packages/EnhancedVolcano.html). The Cytoscape software (http://www.cytoscape.org/) was used for the construction and visualization of the pathway hubs.^21^

### 2.7 Gene set enrichment analysis

GSEA software downloaded from the Broad Institute (http://www.broadinstitute.org/gsea) was utilized to identify gene set variations in two risk groups. In this analysis, the top 25 and bottom 25 patients were compared to identify the most significantly perturbed pathways in the TCGA data set. The genes were preranked based on fold‐change calculated using the DESeq2 algorithm in R and the target gene set was “H: hallmark gene sets” with the number of permutations set at 1000. Enrichment statistic was set to “weighted” and “Signal2Noise” metric was used for ranking genes.

### 2.8 Immune cell infiltration and exhaustion analysis

In this study, the immune cell infiltration landscape of high‐ and low‐risk patients was analyzed using QuantiSeq's computational pipeline.^22^ The *z* scores of exhaustion genes were downloaded for TCGA‐COAD cBioportal.^13^

### 2.9 Statistical analysis

The normalization of raw NanoString data was performed using the nCounter software (NanoString Technologies Inc.). Briefly, the geometric mean of the negative and positive control was used to normalize the expression across all samples. Further normalization was performed using six internal control genes (*ABCF1*, *GUSB*, *HPRT1*, *LDHA*, *POLR1B*, and *RPLO*). Hierarchal clustering of correlation between immune cells and risk groups was generated using Ward's methods. Kaplan–Meier analysis and a log‐rank test was used to compare the survival distribution using the GEPIA portal (TCGA data set) and JMP‐Pro for internal and GEO data sets. During the initial screening, the prognostic gene pairs were iteratively tested for prognostic significance, and H.R and *p* values were derived from the GEPIA portal. Cell plots were generated using JMP‐Pro. In GSEA analysis, the normalized gene enrichment score with >2 value was considered strong. All the statistical analyses were performed using R (version 4.0, R Foundation for Statistical Computing, Vienna, Austria) (http://www.R‐project.org/), JMP‐Pro (version 15.0.0, SAS Institute), and GraphPad Prism (version 9, GraphPad Software). *p* values with ≤0.05 were considered statistically significant.

## 3 RESULTS

### 3.1 Clinical features of CRC patients

The clinicopathological features of the CRC patients included in this study are summarized in Table 1. The clinical variable included age, stage, grade, sex, vital status, metastasis, and chemotherapy. There were several socioeconomic parameters such as ethnicity, alcohol consumption, tobacco consumption, and history of cancer. Clinically, in this data set, most of the patients were >68 years of age (*n* = 65, 69.8%), stage III + IV (*n* = 48, 51.6%), and grade I + II (*n* = 61, 65.5%), and only a subset of patients received chemotherapy (*n* = 31, 33%). Among environmental variables, most patients did not smoke (*n* = 59, 63.4%) or drink alcohol (*n* = 72, 78.2%).

**TABLE 1:** Clinicopathological characteristics of CRC patients

Clinicopathological features		Total = 93 patients	
Age	>68 years	65	69.89%
	<68 years	28	30.10%
Stage	I + II	45	48.30%
	III + IV	48	51.60%
Grade	I + II	61	65.50%
	III	32	34.40%
Sex	Female	53	56.98%
	Male	40	43.01%
Vital status	Alive	33	35.43%
	Dead	60	64.51%
Metastasis	Metastasis	37	40.21%
	No metastasis	55	59.78%
Ethnicity	African American	42	46.66%
	Caucasian	48	53.33%
Alcohol consumption	Alcohol used	20	21.73%
	No alcohol use	72	78.26%
Tobacco consumption	None	59	63.44%
	Smoked	34	36.55%
Cancer history	History of cancer	38	47.50%
	No history of cancer	42	52.50%
Chemotherapy	Chemotherapy administered	31	33.33%
	No chemotherapy	62	66.66%

### 3.2 Development and assessment of immune gene panel

**FIGURE 1:**
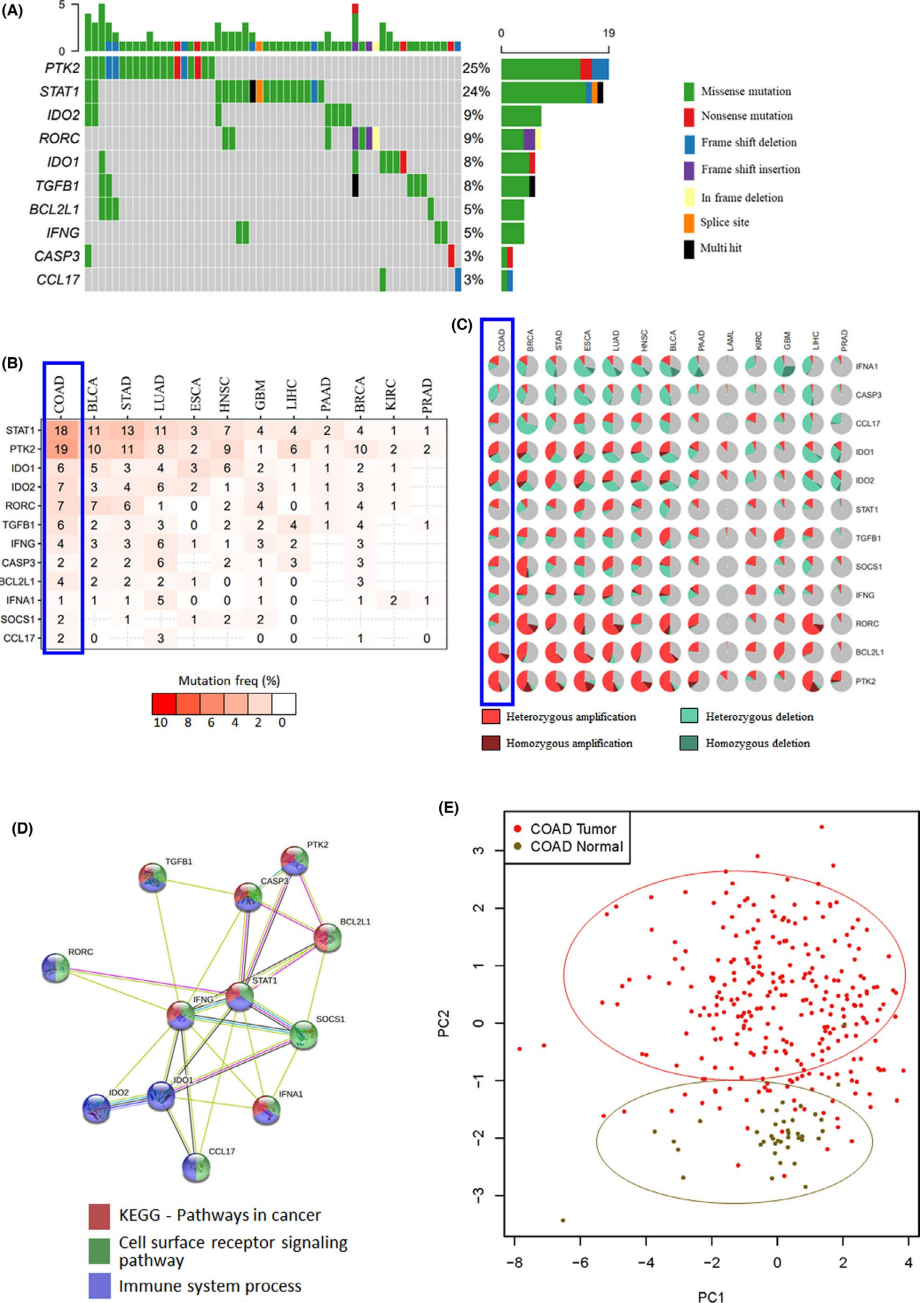
Mutation profiling of immune gene panel in TCGA‐COAD. (A) Waterfall plot showing the landscape of mutations in COAD samples. The tumor mutation burden is depicted as a bar plot above the legend. Different colors depict specific mutation types. (B) SNV (single nucleotide variation) percentage heatmap depicting higher frequency in COAD data set, (C) comparative pie‐plot summary of CNV percentage in major cancer, (D) network analysis of immune genes (E) PCA analysis of 12 genes in normal versus tumor tissue of TCGA‐COAD CRC samples

The genes included in this study were identified from the literature and are known to play a role in the tumor microenvironment (TME) (Table 2). To further characterize their location in the cells of TME, the compartmental distribution of genes was performed using the “genecards” web portal. The distribution of three genes *TGFB1*, *PTK2*, and *BCL2L1* showed distribution in >3 compartments (Table 2). At the clinical level, the genomic analysis identified the highest gene mutations in *PTK2* and *STAT1* with 25% and 24% of TCGA‐COAD patients (Figure 1A). Further, in comparison with other major cancers, the gene set showed a higher mutation frequency (Figure 1B). Copy Number Variation analysis identified the highest heterozygous amplification in *PTK2* and *BCL2L* genes (Figure 1C). To further characterize the interaction of these genes, protein network analysis was performed. It identified close interactions between the genes included in this panel with major pathways related to cancer, cell surface receptor signaling, and immune system processes (Figure 1D). To further characterize the distribution of these genes in cancer, its expression was compared with the normal tissue using PCA (Figure 1E). The variance pattern resulted in a distinct separation of COAD tumors from normal tissue.

**TABLE 2:** Biological roles of immune genes included in this study

Gene	Entrez ID	Confidence value (cell compartment)	Role in tumorigenesis
TGFB1 (transforming growth factor beta 1)	7040	5 (PM, ECM, N, GA)	*TGFB1* plays a critical role in later stages of cancer invasion and metastasis as it promotes features such as EMT^23^
PTK2 (protein tyrosine kinase 2)	5747	5 (PM, CS, N, CY)	Elevated levels of *PTK2* were found to be associated with cancer progression or metastasis^24^
RORC (RAR‐related orphan receptor C)	6097	5 (N)	Th17 expression of *RORC* plays a dual role in tumorigenesis^25^
SOCS1 (suppressor of cytokine signaling 1)	8651	5 (CS, N)	The expression of *SOCS1* was found to decrease with advanced stages and grade^26^
BCL2L1 (BCL2‐like 1)	598	5 (CS, M, N, CY)	The expression of *BCL2L1* is higher in colorectal tumors compared to normal^27^
CASP3 (Caspase 3)	836	5 (N, CY)	Cleaved caspase‐3 has been associated with aggressive cancer^28^
CCL17 (C–C motif chemokine ligand 17)	6361	5 (E)	*CCL17* leads to the migration of human cancer cells^29^
IDO1 (indoleamine 2,3‐dioxygenase 1)	6059	5 (CY)	*IDO1* expression promotes cancer cell survival and proliferation^30^
IDO2 (indoleamine 2,3‐dioxygenase 2)	169355	4 (CY)	*IDO2* plays a role in progression of cancer^31^
IFNA1 (interferon alpha 1)	3439	5 (E)	Interferon therapy perturbs metastasis of colorectal cancer^32^
IFNG (interferon gamma)	3458	5 (E)	Interferon gamma deficiency leads to colorectal cancer progression^33^
STAT1 (signal transducer and activator of transcription 1)	6772	5 (N, CY)	*STAT1* can act as a potential biomarker for early stage colon cancer^34^

*Note*: The cell compartment confidence value ranged from 0 (absence of any evidence) to 5 (highest confidence). The cellular localization of these genes was incorporated from Genecards (https://www.genecards.org/).

Abbreviations: CS, cytoskeleton; CY, cytosol; E, extracellular; ECM, extracellular matrix; GA, golgi apparatus; M, mitochondria; N, nucleus; PM, plasma membrane.

### 3.3 Survival analysis

**FIGURE 2:**
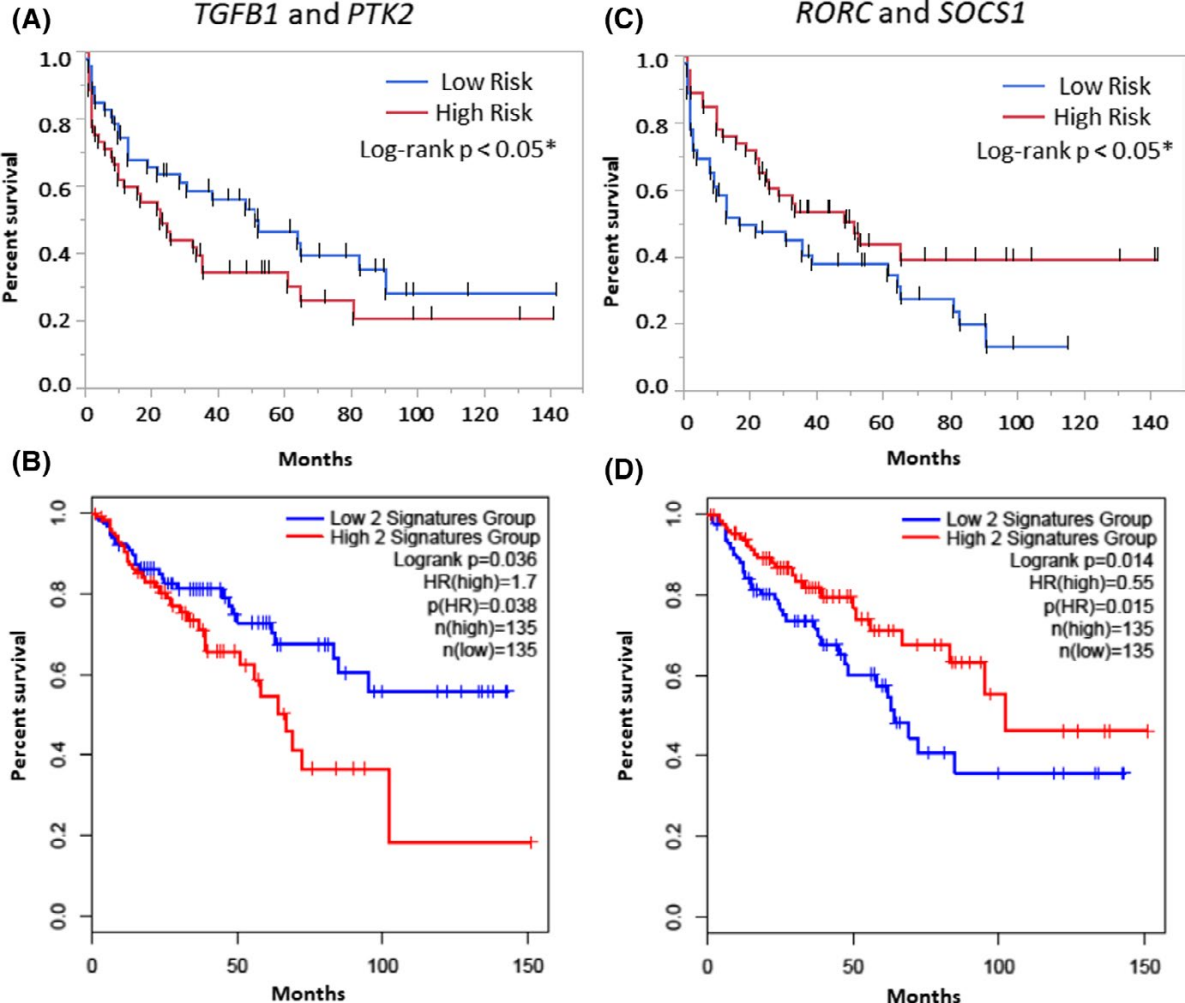
Survival analysis. (A) The combined prognostic score of *TGFB1* and *PTK2* in the internal data set and (B) TCGA data set. (C) The combined prognostic score of *RORC* and *SOCS1* in the internal data set and (D) TCGA data set

**FIGURE 3:**
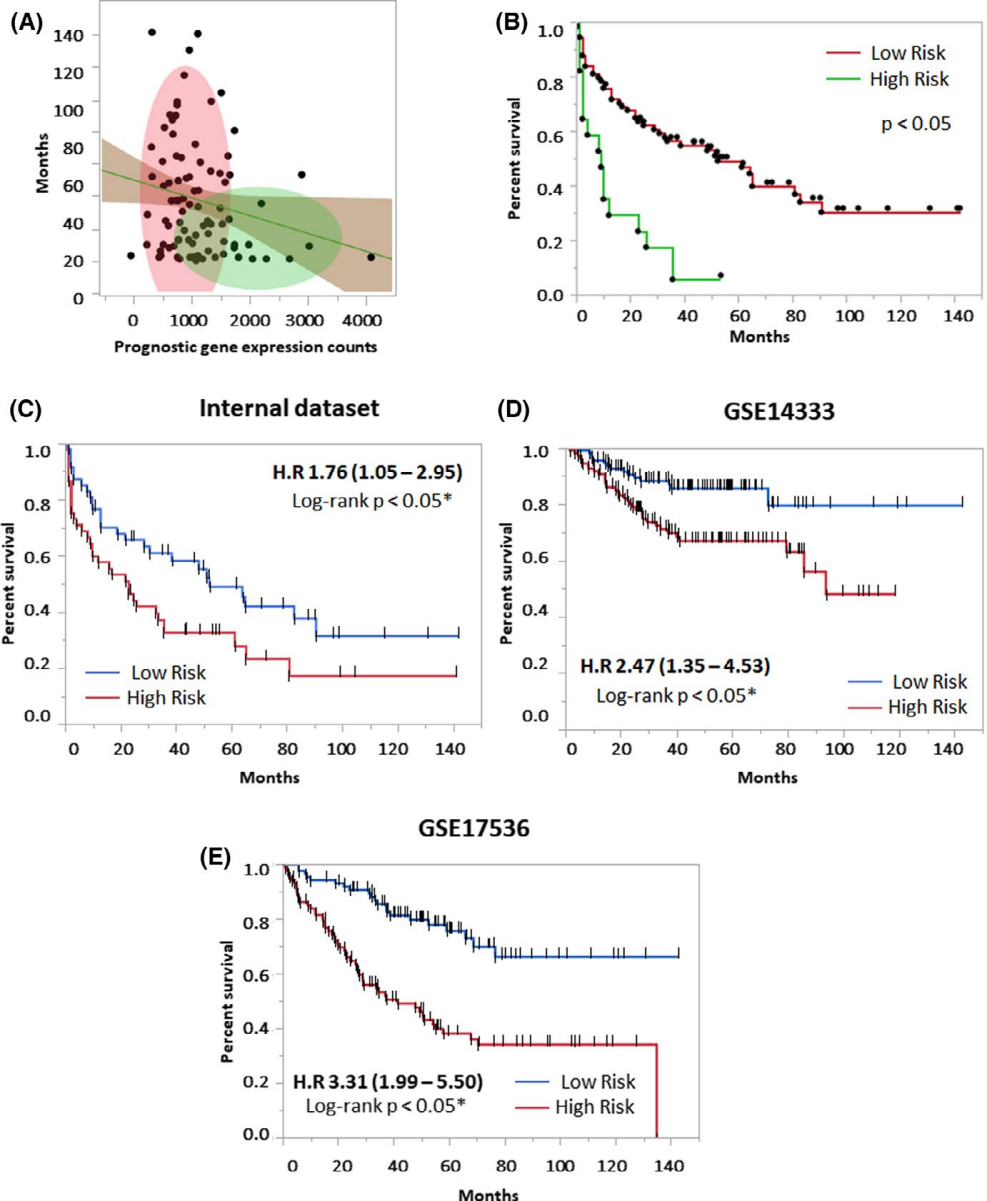
Combined four‐gene prognostic assessment in internal and external data sets. (A) k‐mean clustering of gene expression and overall survival in internal data sets; (B) survival difference between higher and lower gene expression clusters (C) KM estimate based on the four‐gene prognostic score in the internal data set (D) external GEO data set GSE14333, and (E) external GEO data set GSE17536

The survival analysis was performed to identify genes with good and bad prognoses in CRC patients. Iterative screening of gene combinations was performed to identify significant genes (Tables S2 and S3). The combined two‐gene signature (*TGFB1* and *PTK2*) correlated with poor prognosis as its expression was found to be associated with poor overall survival in both internal and TCGA data sets (Figure 2A,B). Further, the combined gene signature of *RORC* and *SOCS1* showed a good prognosis in the internal and TCGA data set (Figure 2C,D). To further evaluate the prognostic distribution, a Cox proportional model was developed to identify the prognostic value of the combined four‐gene signature. In a univariate analysis of the internal data set, the high‐risk group was associated with worse survival (HR: 1.76, 95% CI: 1.05–2.95, *p < 0.02) (Table 3). Other significant variables included higher age (HR: 1.8, 95% CI: 0.98–2.95, *p < 0.02) and higher grade (HR: 2.09, 95% CI: 1.25–3.50, *p < 0.02). The multivariate model based on an internal data set maintained significance in the classification of high‐risk patients (HR: 1.74, 95% CI: 1.02–2.98, *p < 0.04) (Table 3). Further, hazard assessment of the independent gene expression data set validated the prognostic distribution of the four‐gene signature. GSE17536 (177 patients; HR: 3.31, 95% CI: 1.99–5.55, *p < 0.01) and GSE14333 (226 patients, HR: 2.47, 95% CI: 1.35–4.53, *p < 0.01) captured a similar trend of prognostication (Table 4). In the internal data set, k‐mean clustering identified two clusters of patients with a higher four‐gene signature expression, showing worse survival (Figure 3A,B). The KM analysis based on the median‐cutoff of prognostic score classified patients with mortality risk with significance in internal and external data sets (Figure 3C–E).

**TABLE 3:** Univariate and multivariate Cox proportional hazard analysis of the internal data set

Variable	Univariate			Multivariate		
	Hazard ratio	95% CI	*p* value	Hazard ratio	95% CI	*p* value
Combined four‐gene signature (high, low)	1.76	1.05–2.95	0.02*	1.74	1.02–2.98	0.04*
Age (>68 years, <68 years)	1.8	0.98–3.30	0.05	1.72	0.90–3.26	0.09
Stage (I + II, III + IV)	0.86	0.51–1.44	0.51	1.12	0.62–2.00	0.7
Sex (male, female)	1	0.60–1.68	0.97	1.06	0.63–1.78	0.82
Chemotherapy (yes, no)	0.94	0.54–1.62	0.83	1.05	0.30–1.10	0.09
Grade (I + II, III)	2.09	1.25–3.50	0.004*			
Metastasis (metastasis, no metastasis)	0.82	0.48–1.41	0.48			
Ethnicity (African American, Caucasian)	1.42	0.85–2.37	0.17			
Alcohol consumption (yes, no)	1.12	0.61–2.08	0.71			
Tobacco consumption (yes, no)	0.79	0.46–1.34	0.38			
Cancer history (yes, no)	0.59	0.33–1.05	0.07			

* *p* ≤ 0.05

**TABLE 4:** Validation of prognostic scores in independent GEO data set

Variable	GSE 17536			GSE 14333		
	Hazard ratio	95% CI	*p* value	Hazard ratio	95% CI	*p* value
Combined four‐gene signature (high, low)	3.31	1.99–5.55	0.01*	2.47	1.35–4.53	0.01*
Age (>50 years, <50 years)	0.77	0.36–1.61	0.49	0.68	0.33–1.41	0.33
Sex (male, female)	1.1	0.69–1.75	0.67	0.9	0.62–1.92	0.73
Chemotherapy (yes, no)				1.89	1.08–3.29	0.02*
Stage (III + IV, I + II)	4.22	2.3–7.46	0.01*			
Grade (III, II + I)	2.19	1.25–3.82	0.01*			
Ethnicity (African American, Caucasian)	2.26	0.97–5.25	0.05*			

* *p* ≤ 0.05

### 3.4 Differential expression of genes

**FIGURE 4:**
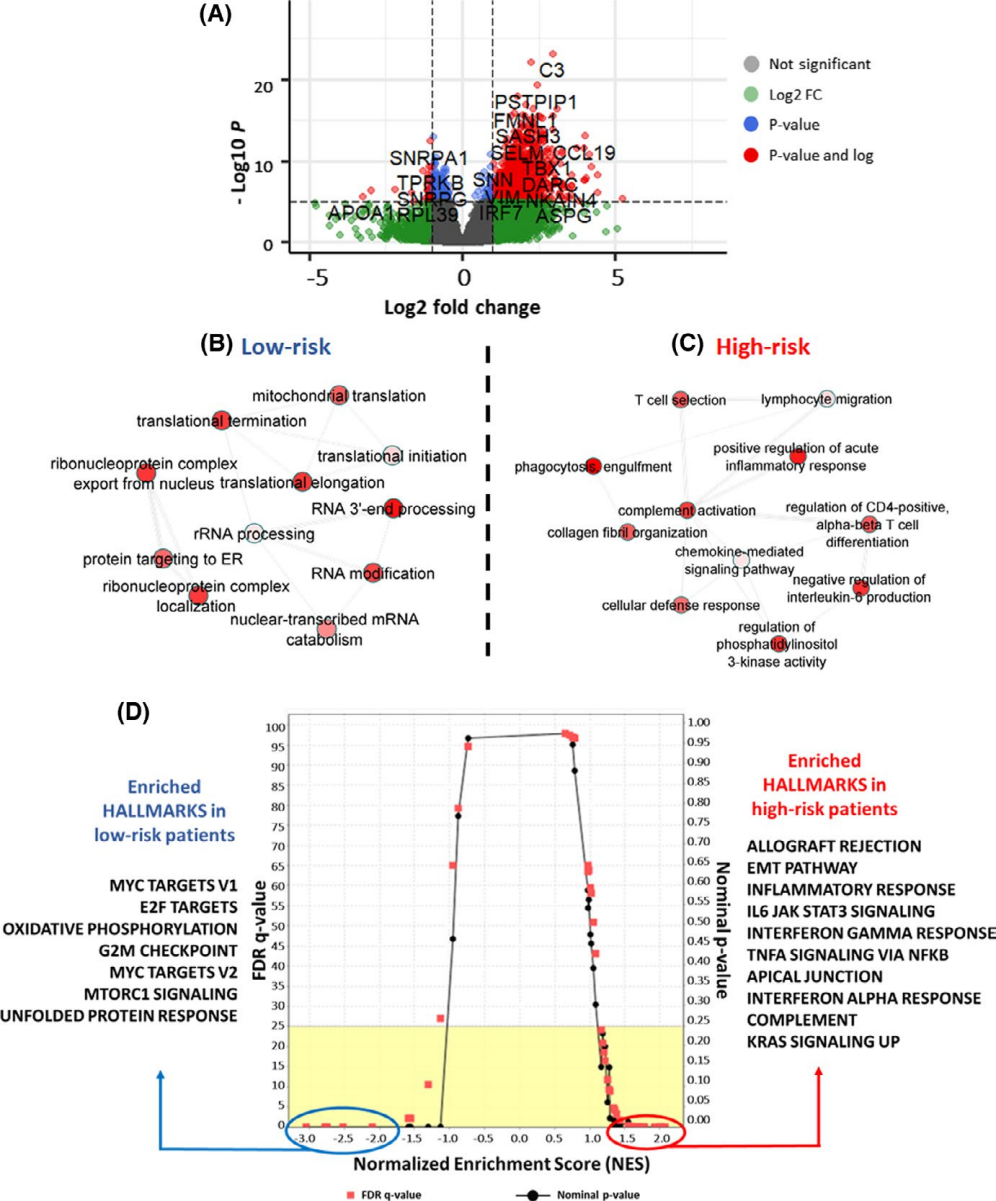
(A) Volcano plot depicting differential expression of genes in high‐risk and low‐risk CRC patients in TCGA data set. (B) GO term enrichment in the low‐risk group. (C) GO term enrichment in the high‐risk group and (D) differential enrichment of hallmarks in cancer in two risk groups

Differential expression analyses between high‐ and low‐risk patient’ groups were performed using DEseq2 analysis (Table S4). Among 20,501 protein‐coding genes, 561 genes were upregulated >2‐fold in high‐risk patients compared with the lower‐risk group. In the low‐risk group, a total of 93 genes were upregulated >2‐fold compared with the high‐risk group. A volcano plot of differentially expressed genes between high‐risk and low‐risk patients is depicted in Figure 4A. The enriched gene ontology terms in the low‐risk group included pathways such as translation, RNA processing, translation, and mitochondrial translocation (Figure 4B). The enriched pathways in the high‐risk group were immune‐related pathways such as T cell migration, complement and defense response, and acute‐phase response pathways (Figure 4C). Further, GSEA identified inflammatory gene expression and EMT pathways compared with MYC and E2F target genes enrichment (Figure 4D).

### 3.5 Gene set enrichment analysis

**FIGURE 5:**
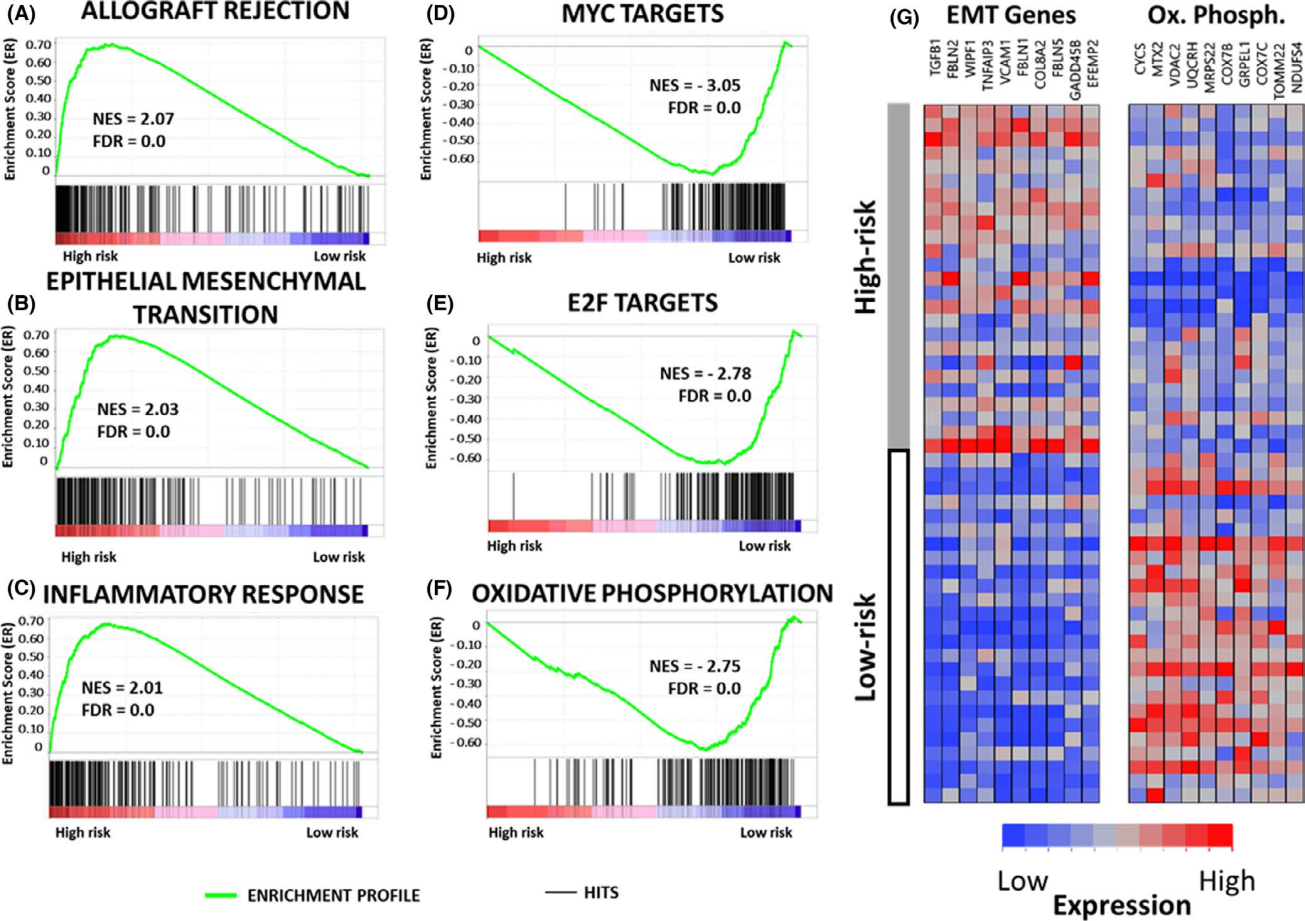
Gene set enrichment analysis based on risk stratification in TCGA data set. In the high‐risk group, enriched pathways included (a) allograft rejection, (B) epithelial–mesenchymal transition, and (C) inflammatory response. In the low‐risk group, (D) MYC targets, (E) E2F targets, and (F) oxidative phosphorylation were enriched. (G) Comparative analysis of the high‐ and low‐risk groups, depicting differential expression of genes in key pathways

To analyze gene perturbation in two risk clusters, GSEA results were analyzed through normalized enrichment scores (NESs). The HALLMARK pathways with the highest NES in the high‐risk group were allograft rejection (NES = 2.07, FDR ≤ 0.001), epithelial–mesenchymal transition (NES = 2.03, FDR ≤ 0.001), and inflammatory response (NES = 2.01, FDR ≤ 0.001) (Figure 5A–C). Interestingly, patients in the low‐risk category showed enrichment in MYC targets (NES = −3.05, FDR ≤ 0.001), E2F targets (NES = −2.78, FDR ≤ 0.001), and oxidative phosphorylation (NES = −2.75, FDR ≤ 0.001) (Figure 5D–F). Cell plot analysis captured the variation of gene expression in the two risk groups (Figure 5G).

### 3.6 Infiltration of immune cells and exhaustion analysis

**FIGURE 6:**
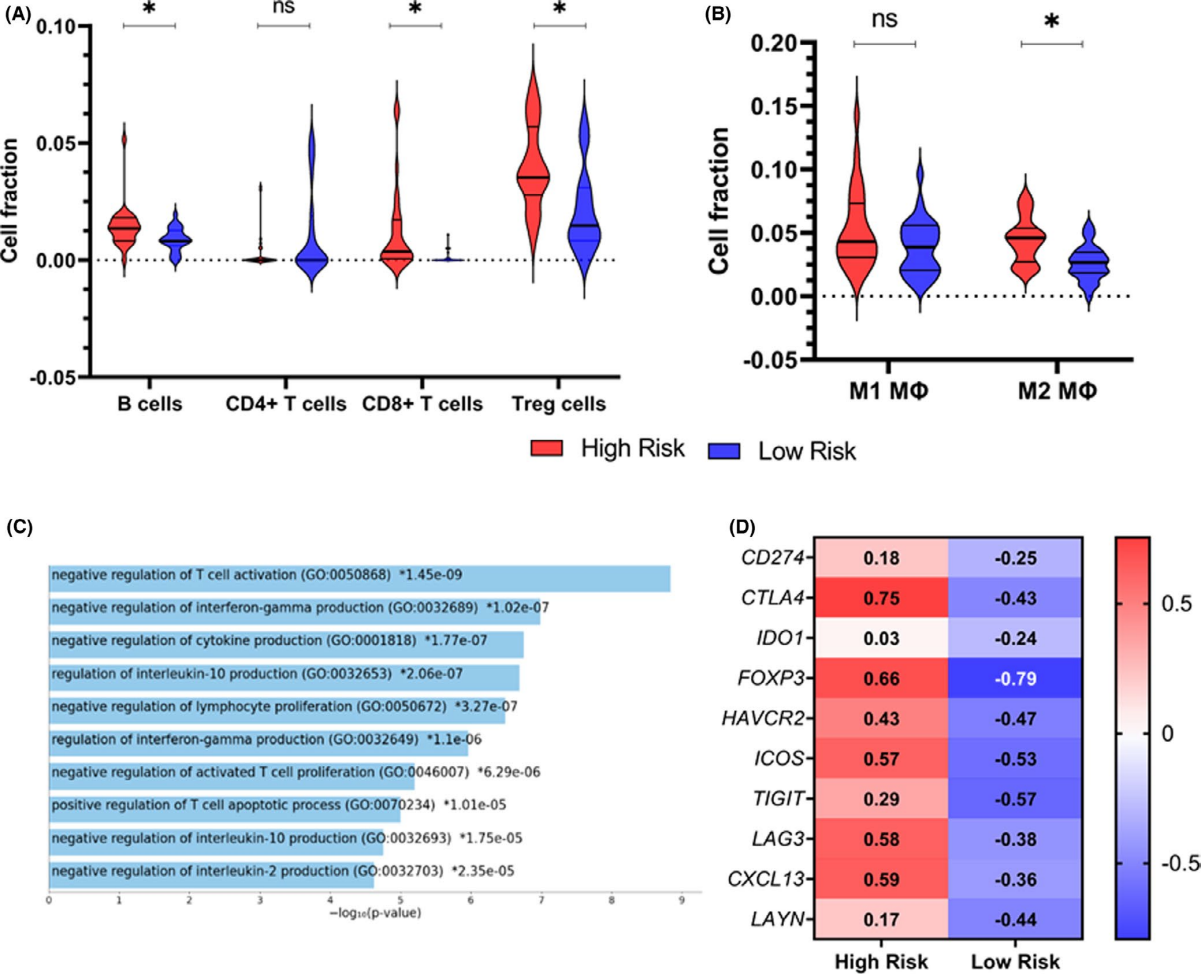
(A) Differential infiltration of immune cells in high‐risk and low‐risk groups of TCGA data set. (B) Infiltration of macrophages. (C) Pathway clustering of exhaustion genes. (D) *z*‐score of exhaustion genes in the two risk groups

To analyze the distribution of immune cells in these two risk groups, the Quantiseq algorithm was utilized. Patients at high risk showed an abundance of B cells, CD8^+^ T cells, and T‐reg cells (Figure 6A). Among macrophages, M2 macrophages showed a significant association with the high‐risk group (Figure 6B). To further evaluate the immunosuppressive microenvironment, the expression of 10 immune gene exhaustion genes was quantified in both the high‐risk and low‐risk groups. These genes were mostly involved in the negative regulation of T cells (Figure 6C). Interestingly, patients in the high‐risk group showed higher expression (higher *z*‐score) of all the immune exhaustion genes (Figure 6D).

## 4 DISCUSSION

CRC is a complex disease with a heterogeneous tumor microenvironment that plays a critical role in disease progression.^35^ The advancements in transcriptomics have led to the identification of several gene expression‐based classification methods for colorectal cancer.^36^, ^37^, ^38^, ^39^ Recently, an inflammatory gene module of 14 genes has been identified as prognostic biomarkers for colorectal cancer.^40^ Despite several advances, there is limited progress in the validation and characterization of biological networks in high‐risk populations. In this study, we have identified a four‐gene prognostic signature (*TGFB1*, *PTK2*, *RORC*, and *SOCS1*) using FFPE tissue and assessed its distribution in public data sets. TGFB1 is a ligand secreted by several cells in the tumor microenvironment and plays a critical role in lung carcinoma.^41^ Its activity affects cell proliferation, differentiation, metastasis, cell adhesion, and resistance to drugs.^42^, ^43^ Further, TGFB expression in the tumor microenvironment fuels transformation of normal fibroblasts into cancer‐associated fibroblasts (CAFs) in CRC.^44^ Also, the TGFB pathway is a key player in EMT, tumor invasion, metastasis to the liver, and angiogenesis.^43^ In a recent study, the TGFB‐activating‐like gene phenotype showed worse survival compared with the TGFB‐inactivating‐like signature.^45^ In a recent study on the serum of CRC patients, TGFB1 levels were found to be higher in untreated patients compared with patients under chemotherapeutic treatment. In this study, TGFB1 signaling was found to be critical for metastasis and stromal cell independence.^46^ A second gene, protein tyrosine kinase 2 (PTK2) is a member of the nonreceptor tyrosine kinase and regulates cell proliferation, migration, and invasion.^47^ Higher PTK2 expression is associated with cancer progression, metastasis, and poor overall survival.^48^ In a network analysis of different genes, PTK2 was identified as an inflammation‐related gene signature in CRC.^40^ Further, PTK2 activation confers adaptive resistance to chemotherapy in CRC.^49^ The third gene in our set, RORC is a member of the nuclear orphan receptor family and is involved in cell growth, metastasis, and resistance to chemotherapy in various tumors. In bladder cancer, higher RORC expression led to suppression of cell growth, glucose metabolism, and negatively regulated growth of PD‐L1.^50^ Further, it has been shown that the tissue microenvironment plays a critical role in tumor‐suppressive effects of immune cells.^51^ SOCS1 negatively regulates JAK–STAT signaling pathways and is involved in the inhibition of inflammation. In an earlier study, SOCS1 was identified as a tumor suppressor in certain colorectal cancer patients.^52^ SOCS1 has also been shown to have a role in tumor progression in CRC.^53^ It has been previously reported that SOCS1‐based suppression of Src activity led to downregulation of the EMT pathway.^54^ Thus, the function of SOCS1 can be either tumor‐suppressive or oncogenic and is dependent on tissue *milieu*.^55^

In this study, network analysis identified significant upregulation of immune‐related and EMT pathways in high‐risk patients. EMT progression has been shown to play a central role in tumor initiation and metastasis of CRC.^56^ In low‐risk CRC patients, cancer progression was found to depend upon oxidative phosphorylation and MYC and E2F targets. In CRC tumors, OXPHOS has been shown to promote tumor progression, metastasis, and resistance to drugs.^57^ Further, our study identified higher infiltration of T‐regulatory cells and M2 macrophages in the high‐risk group. Previous analysis in CRC patients has associated infiltration of T‐regulatory cells and M2 macrophages with poor prognosis.^58^ Additionally, we identified higher infiltration of B cells, CD8+ T cells, and immune exhaustion genes in high‐risk patients. Several studies have documented the upregulation of immune exhaustion in CRC tumors.^59^, ^60^

In a recent study on metastatic CRC mouse model, TGFB blockade led to susceptibility to anti‐PD1‐PD‐L1 therapy.^61^ Although anti‐TGFB has shown improved efficacy in PD‐1/PD‐L1, its clinical assessment remains under study.^62^ Similarly, to counter the effects of PTK2 overexpression, PTK2 inhibitors are being explored in several clinical trials.^63^, ^64^ On the other hand, RORC was included in an immune panel that can act as a predictive biomarker for determining the efficacy of anti‐PD‐1 treatment in NSCLC patients.^65^ In this study, we have correlated gene expression of inflammatory, EMT pathways, and immune exhaustion genes with high‐risk patients that can provide insights for stratification of patients for personalized therapies. This strategy can also improve the efficacy of current treatment strategies and can lead to better outcomes for CRC patients.

## CONFLICT OF INTEREST

The authors declare that they have no competing interests.

## AUTHORS' CONTRIBUTION

Pankaj Ahluwalia and Ravindra Kolhe conceived, designed, and wrote the first draft. Pankaj Ahluwalia, Ashis K. Mondal, Meenakshi Ahluwalia, Nikhil S. Sahajpal, Kimya Jones, and Yasmeen Jilani performed the experiments. Meenakshi Ahluwalia, Gagandeep K. Gahlay, Amanda Barrett, Vamsi Kota, and Amyn M. Rojiani helped in the manuscript and data review. Ravindra Kolhe supervised and provided financial resources for the study. All authors approved the final version of the manuscript.

## ETHICS APPROVAL

The study was approved by the Institutional Review Board (IRB‐HAC # 611298) of Augusta University.

## Supporting information


Table S1

Table S2

Table S3

Table S4

## Data Availability

The data sets used and/or analyzed in the current study are available from the corresponding author on reasonable request.
